# Supplementary material for: 1-Piperidine Propionic Acid as an Allosteric Inhibitor of Protease Activated Receptor-2
Source: Pharmaceuticals (Basel). 2023 Oct 18;16(10):1486. doi: 10.3390/ph16101486 (PMC10610151; doi:10.3390/ph16101486)
Supplement: Supplementary file 1 [file pharmaceuticals-16-01486-s001.zip › Chinellato_Supplementary Figure S1 Rev2.pdf]

Supplementary Figures

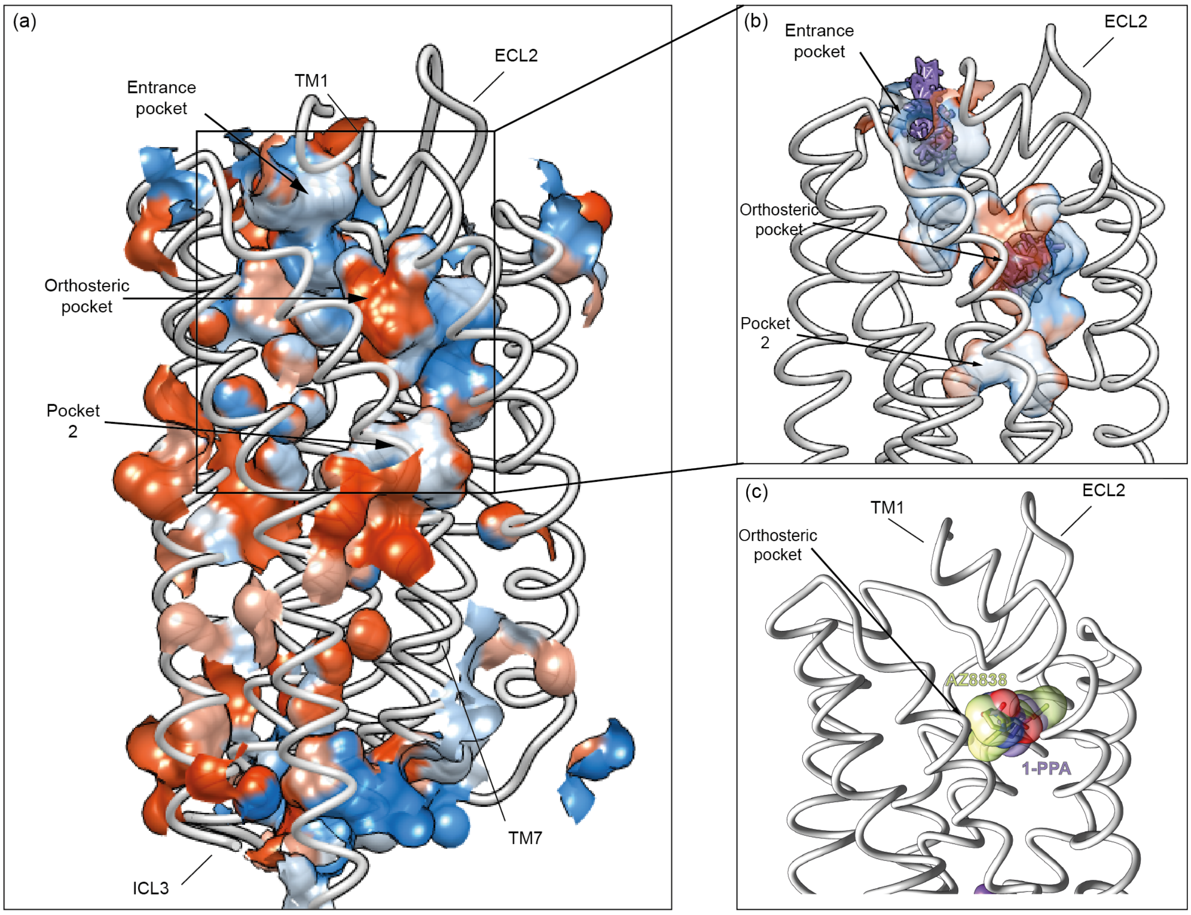

Figure S1. Binding pockets and Docking of 1-PPA and AZ8838. (**a**) *wt*PAR2 model of the transmembrane domain (Val61-Arg362) in white. Surface with charges-based coloring (red: negatively charged regions, blue: positively charged region); (**b**) Zoom of the biggest pockets showing the overlay of the docking analysis with 1-PPA (in purple); (**c**) Zoom on the orthosteric pocket showing the overlay of 1-PPA (purple) with AZ8838 (green).
