# Supplementary material for: 1-Piperidine Propionic Acid as an Allosteric Inhibitor of Protease Activated Receptor-2
Source: Pharmaceuticals (Basel). 2023 Oct 18;16(10):1486. doi: 10.3390/ph16101486 (PMC10610151; doi:10.3390/ph16101486)
Supplement: Supplementary file 1 [file pharmaceuticals-16-01486-s001.zip › Chinellato_Supplemetary Figure S2 Rev2.pdf]

Supplementary Figures

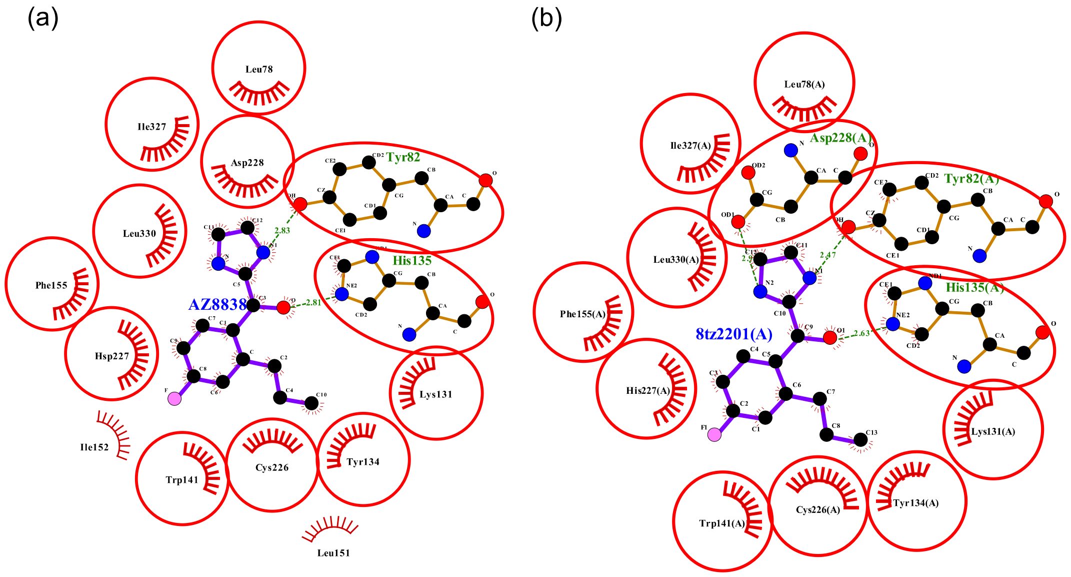

Figure S2. LigPlot comparison of PAR2 residues interacting with AZ8838 in 5NDD structure and docking simulation. (**a**) schematic representation of identified interactions between AZ8838 and PAR2 from SwissDock binding mode identification; (**b**) schematic representation of identified interactions between AZ8838 and PAR2 in 5NDD crystallographic structure.
