# Supplementary material for: 1-Piperidine Propionic Acid as an Allosteric Inhibitor of Protease Activated Receptor-2
Source: Pharmaceuticals (Basel). 2023 Oct 18;16(10):1486. doi: 10.3390/ph16101486 (PMC10610151; doi:10.3390/ph16101486)
Supplement: Supplementary file 1 [file pharmaceuticals-16-01486-s001.zip › Chinellato_Supplemetary Figure S3 Rev2.pdf]

Supplementary Figures

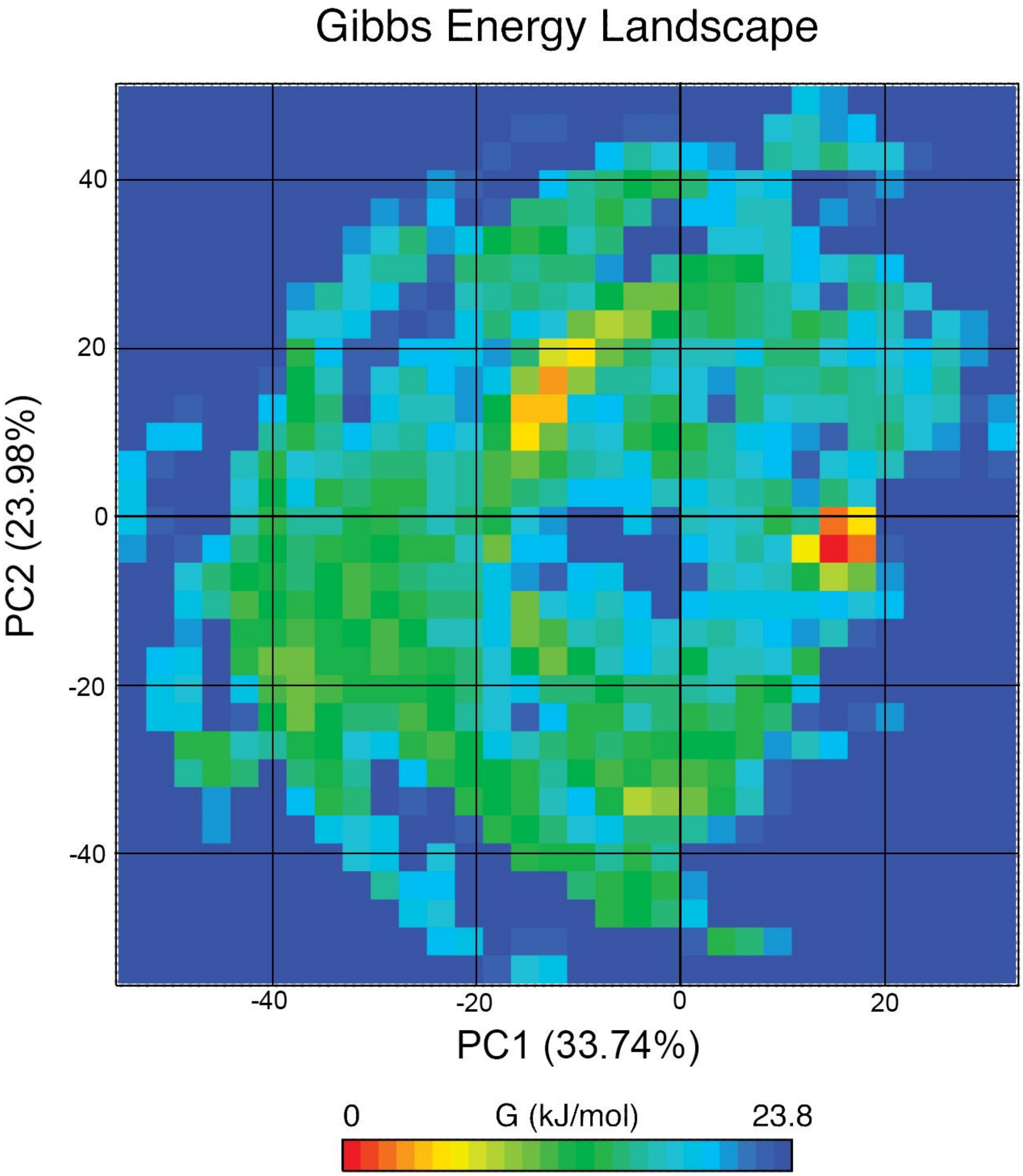

Figure S3. Supplementary figure 2. Motions PCA of the Par2-PPA complex. States of the protein complex were plotted against the first two eigenvectors (explaining 33,74% and 23,98% of variance respectively) and clustered based on density. Regions with lower relative free energy are depicted in red, whereas higher energies are depicted in blue. The final state of the simulation analyzed throughout the manuscript lies in the lowest energy region and is thus the most favorable conformation between the sampled one
