# Supplementary material for: 1-Piperidine Propionic Acid as an Allosteric Inhibitor of Protease Activated Receptor-2
Source: Pharmaceuticals (Basel). 2023 Oct 18;16(10):1486. doi: 10.3390/ph16101486 (PMC10610151; doi:10.3390/ph16101486)
Supplement: Supplementary file 1 [file pharmaceuticals-16-01486-s001.zip › Chinellato_Supplemetary Figure S4 Rev2.pdf]

Supplementary Figures

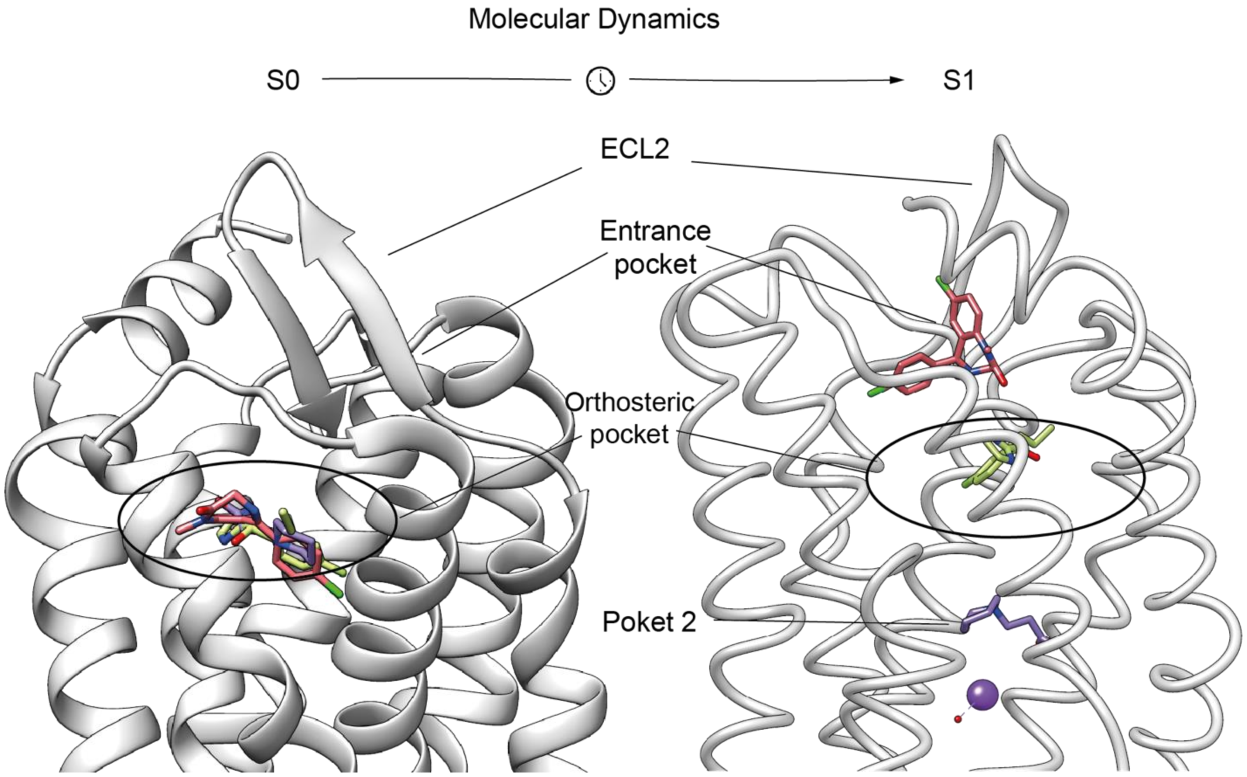

Figure S4. Movements of compounds during MD simulations. *wt*PAR2 represented in white ribbons and *wt*PAR2 white licorice, respectively, for the Initial State (S0) and Final State (S1) were obtained from the Molecular Dynamics simulations. Orthosteric pocket is circled in both the structure. AZ8838 (green) shows no movement from the orthosteric pockets throughout the simulation. 1-PPA (purple) and Ro5-4864 (pink) move from the pocket with divergent directions: 1-PPA gets engulfed in the allosteric pocket, otherwise Ro5-4864 moves towards the extracellular space.
